# Preparation and Application of Nb_2_O_5_ Nanofibers in CO_2_ Photoconversion

**DOI:** 10.3390/nano11123268

**Published:** 2021-12-01

**Authors:** A. C. F. Prado, J. O. D. Malafatti, J. A. Oliveira, C. Ribeiro, M. R. Joya, A. P. Luz, E. C. Paris

**Affiliations:** 1 Nanotechnology National Laboratory for Agriculture (LNNA), Embrapa Instrumentação, XV de Novembro st., 1452, São Carlos 13560-970, SP, Brazil; anacfprado@gmail.com (A.C.F.P.); jmalafatti@hotmail.com (J.O.D.M.); jessicarianeoliveira@hotmail.com (J.A.O.); caue.ribeiro@embrapa.br (C.R.); 2 Graduate Program in Materials Science and Engineering (PPGCEM), Department Materials Engineering, Federal University of São Carlos, Rodovia Washington Luiz, Km 235 SP-310, São Carlos 13565-905, SP, Brazil; anapradol@gmail.com; 3 Department Materials Science and Engineering (PPGCEM), Federal University of São Carlos, Rodovia Washington Luiz, Km 235 SP-310, São Carlos 13565-905, SP, Brazil; 4 Department of Chemistry, Federal University of São Carlos, Rod. Washington Luís, Km 235, São Carlos 13565-905, SP, Brazil; 5 Department of Chemical Engineering, Federal University of São Carlos, Rod. Washington Luís, Km 235, São Carlos 13565-905, SP, Brazil; 6 Departamento de Física, Facultad de Ciencias, Universidad Nacional de Colombia-Bogotá, Carrera 30 Calle 45-03, Bogotá 111321, Colombia

**Keywords:** Nb_2_O_5_, CO_2_ conversion, photocatalysis, electrospinning, nanofibers

## Abstract

Increasing global warming due to NO${}_{x}$, CO${}_{2}$, and CH${}_{4}$, is significantly harming ecosystems and life worldwide. One promising methodology is converting pollutants into valuable chemicals via photocatalytic processes (by reusable photocatalysts). In this context, the present work aimed to produce a Nb${}_{2}$O${}_{5}$ photocatalyst nanofiber system by electrospinning to convert CO${}_{2}$. Based on the collected data, the calcination at 600 ${}^{\circ }$C for 2 h resulted in the best condition to obtain nanofibers with homogeneous surfaces and an average diameter of 84 nm. As a result, the Nb${}_{2}$O${}_{5}$ nanofibers converted CO${}_{2}$ mostly into CO and CH${}_{4}$, reaching values around 8.5 μmol g${}^{-1}$ and 0.55 μmol g${}^{-1}$, respectively.

## 1. Introduction

Various methodologies have been developed to remove pollutants from different types of media, and to address growing environmental quality concerns. In this context, heterogeneous photocatalysis is receiving attention due to its ability to remove and degrade contaminants, recover the photocatalyst, and reuse it in new cycles [1,2]. Specifically, CO${}_{2}$ photoreduction is receiving attention because of the urgent demand to decrease greenhouse gases and diminish their harmful environmental impacts [3]. Photocatalysis is based on complex catalytic reactions that enable the formation of specific products, such as methanol, methane, carbon monoxide, formic acid, and others [4]. Different catalyst systems have been studied, such as TiO${}_{2}$[5], ZnO [6], and WO${}_{3}$ [7], in order to improve the photocatalytic stability, production rate, and selectivity.

Zeng et al. [8] investigated the Cu${}_{2}$O nanowire incorporation in titanium carbide (Ti${}_{3}$C${}_{2}$). These authors have confirmed the enhancement efficiency in CO${}_{2}$ to methanol conversion, causing an increase in the production, around 8.25 times compared to the free Cu${}_{2}$O nanowires. This performance improves the charge carrier transport and reduces the band gap (from 2.2 to 2.02 eV), optimizing the light absorption capacity and modifying the charge recombination processes. Ye et al. [9] designed bismuth-based heterostructured nanotube photocatalysts for CO${}_{2}$ photoconversion. Among the as-synthesized BiOX-type heterostructures (with X equal to Cl, Br, or I), the BiOI sample showed the best performance under visible irradiation due to the smallest band gap (1.7–1.8 eV), favoring the CO${}_{2}$ photoreduction to CO and CH${}_{4}$ (19.82 μmol g${}^{-1}$h${}^{-1}$ and 0.22 μmol g${}^{-1}$h${}^{-1}$, respectively).

Niobium pentoxide (Nb${}_{2}$O${}_{5}$) is a semiconductor that is receiving attention because it possesses similar features to TiO${}_{2}$ and has a promising performance when applied in CO${}_{2}$ photoconversion [10,11,12]. Silva et al. [12] studied the photocatalytic activity of Nb${}_{2}$O${}_{5}$ particles after surface modifications with peroxo groups. As a result, the selectivity and photocatalytic activity were related to the surface acidity of the nanoparticles. Thus, high surface acidity promoted CO${}_{2}$ conversion into CO, HCOOH, and CH${}_{3}$COOH, whereas low acidity induced mainly CH${}_{4}$ formation. Although niobium-based materials are promising alternatives to be applied in CO${}_{2}$ photoconversion, Nb${}_{2}$O${}_{5}$ ceramic nanofibers used for this application were not found in the literature.

Nanofibers are used to apply semiconductors for photocatalysis to degrade pollutants [13,14,15], due to porosity control and homogeneous diameter distributions that enhance the availability of catalytic sites. Deng et al. [13] obtained Ni-NiS/C/ZnO ceramic nanofibers (400 nm diameter) for the CO${}_{2}$ photocatalytic reduction. The authors observed that carbon addition increased the CO and CH${}_{4}$ photocatalytic conversion to 5.86 and 1.14 μmol g${}^{-1}$h${}^{-1}$, respectively. This result is attributed to the catalyst’s ability to increase the system charge separation efficiency.

Xu and collaborators [14] synthesized CuInS${}_{2}$/TiO${}_{2}$ nanofibers (150 nm diameter) by electrospinning and hydrothermal treatment for CO${}_{2}$ photoreduction. As for the result—the photoreduction promoted the formation of CH${}_{4}$ (2.5 μmol g${}^{-1}$h${}^{-1}$) and CH${}_{3}$OH (0.86 μmol g${}^{-1}$h${}^{-1}$) products. The authors also evaluated that CuInS${}_{2}$ increasing enabled more electrons generated and/or transferred to react with CO${}_{2}$ molecules, and improved the CH${}_{4}$ formation. In addition, Kang et al. [15] obtained g-C${}_{3}$N${}_{4}$/TiO${}_{2}$ nanofibers with significant CO${}_{2}$ conversion to CO (5.18 μmol g${}^{-1}$) and CH${}_{4}$ (1.65 μmol g${}^{-1}$), indicating that oxygen vacancies in the disordered surface accelerated charge separation and transport. In this sense, a semiconductor in nanofiber form has shown efficient morphology in being applied to pollutant photoconversion [16,17], as evidenced for CO${}_{2}$ photoreduction.

Recent literature [18,19] has focused on producing niobium-based fibers for photodegradation of the organic pollutants, using polyvinylpyrrolidone (PVP) as a polymeric precursor. The results indicate that the pseudohexagonal TT-Nb${}_{2}$O${}_{5}$ phase showed greater efficiency, more than 62% degradation. Nevertheless, only a few works [20,21,22,23] have been published regarding the performance of Nb${}_{2}$O${}_{5}$ semiconductor fibers.

To the best of our knowledge, no investigations have been carried out considering polyvinyl alcohol (PVA), which is highly soluble in water, as a polymeric precursor for the Nb${}_{2}$O${}_{5}$ fibers synthesis. Furthermore, despite works related to the Nb${}_{2}$O${}_{5}$ systems, there are still critical approaches regarding its role and performance in the CO${}_{2}$ photoreduction process. Based on these aspects, the present work addressed the Nb${}_{2}$O${}_{5}$ ceramic nanofiber synthesis and characterization for CO${}_{2}$ photoreduction, considering the electrospinning method and using polyvinyl alcohol as a precursor under different annealing conditions.

## 2. Materials and Methods

### 2.1. Synthesis of Nb${}_{2}$O${}_{5}$ Fibers

For producing ceramic fibers, polyvinyl alcohol (PVA, Mw 50000, Sigma Aldrich, 99.9% purity) and niobium ammonia oxalate (OAN, Mw 353.02, donated by CBMM-Brazil) were used. First, PVA was solubilized in deionized water to obtain a 20% (w V${}^{-1}$) concentration solution. In parallel, the OAN precursor salt was solubilized in deionized water to obtain a concentration of 40% (w w${}^{-1}$). The solutions were then mixed and homogenized under magnetic stirring for about 30 min. Subsequently, the solution was subjected to the electrospinning method in fixed conditions: ejection rate of 0.7 mLh${}^{-1}$, work distance of 10 cm, and electrical voltage of 20 kV. Finally, the obtained fibers were subjected to thermal treatment based on the literature [24], varying the temperature from 400 ${}^{\circ }$C to 900 ${}^{\circ }$C for 2 h, and a heating rate of 1 or 10 ${}^{\circ }$C min${}^{-1}$.

### 2.2. Characterization

Thermogravimetric analysis (TGA) was performed on fiber precursors to verify the events of polymer matrix degradation and mass loss. The equipment used was TA Instruments, model Q500. The fibers were heated at a temperature between 30 and 900 ${}^{\circ }$C, with a heating rate of 10 °C min${}^{-1}$ and a synthetic air atmosphere with a flow rate of 10 mL h${}^{-1}$. X-ray diffraction (XRD) measurements were used to identify the crystalline structural phase in a Shimadzu XRD 6000 diffractometer, with 30 kV of voltage and 30 mA of current, using Cu Kα radiation (λ = 1.5488 Å). The structural phase quantification was made from the Rietveld refinement by GSAS-EXPGUI software, using the micrometric yttrium oxide pattern, as with instrumental parameters acquired by the Le Bail method. The scanning electron microscopy (SEM) technique allowed the obtainment of Nb${}_{2}$O${}_{5}$ fiber images that provided information about shape, diameter, and distribution, using a JEOL® model 6701F microscope. In addition, the average diameters of the samples were measured with the aid of Image J software.

### 2.3. Immobilization of Nb${}_{2}$O${}_{5}$ Ceramic Fibers

The selected photocatalytic materials were immobilized on glass slides (2 × 2 cm) previously cleaned in a sonicator bath in Extran, water, acetone, ethanol, and water sequentially. From then, the fibers were added to a beaker containing 5 mL of ethanol for dispersion in a thermostatic bath at room temperature (25 ${}^{\circ }$C) for 30 min. After this procedure, the glass slide was placed over a heating plate kept at 50 ${}^{\circ }$C. Finally, the suspension was slowly dropped over the substrate surface until a film formed along the entire glass slide length.

### 2.4. Evaluation of CO${}_{2}$ Gaseous Photoconversion

The immobilized photocatalysts were inserted into the cavity of a stainless steel reactor. The system was purged with an ultra-pure gas containing CO${}_{2}$ and water vapor for 20 min before the experiment. Afterward, the reactor was sealed and exposed to UV-C irradiation (TUV Philips 18 W mercury lamp, 254 nm) for 6 h at room temperature. Aliquots (300 μL) were removed every hour and analyzed in a gas chromatography (GC), CG Varian model CP-3800 equipped with a thermal conductivity detector (TCD), and a flame ionization detector (FID), using a column (HayeSep N (0.5 m × 1.8″)) with a flow rate of 30 mL min${}^{-1}$ for H${}_{2}$, 300 mL min${}^{-1}$ for air, and 30 mL min${}^{-1}$ for N${}_{2}$, using argon as carrier gas. The injector temperature was set at 150 ${}^{\circ }$C, while the TCD and FID detector temperatures were 200 ${}^{\circ }$C and 150 ${}^{\circ }$C, respectively.

## 3. Results

### 3.1. Optimization of the Nb${}_{2}$O${}_{5}$ Ceramic Fibers

**Figure 1:**
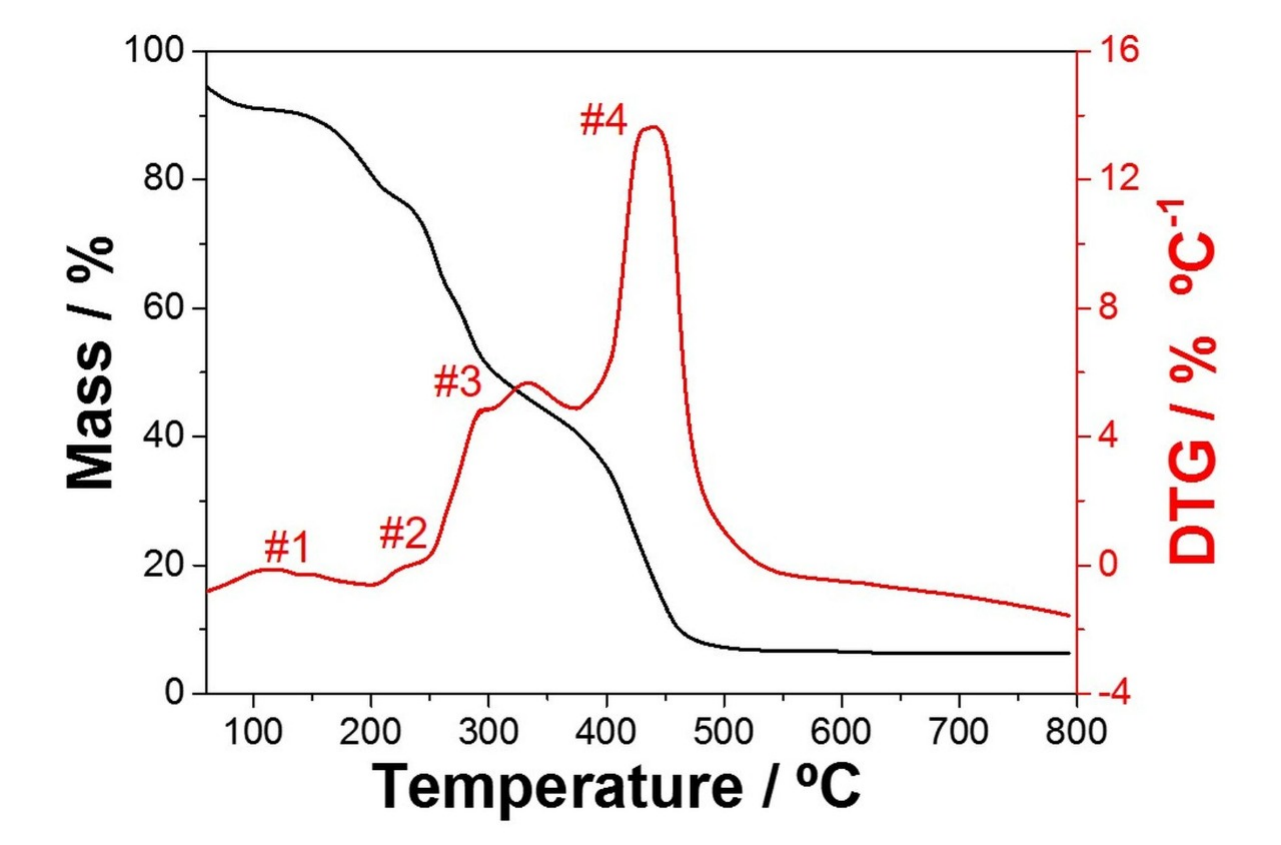
Thermogravimetric profile of the evaluated PVA:OAN fibers.

The thermogravimetric analysis indicated the loss of mass and the polymeric degradation events when the fibers were subjected to thermal heating. Figure 1 shows the PVA fiber degradation behavior in the range from 70 ${}^{\circ }$C to 800 ${}^{\circ }$C. As observed, the prepared polymeric fibers showed a mass loss of up to 480 ${}^{\circ }$C. The thermal event identified at 100 to 150 ${}^{\circ }$C was due to the sample adsorbed water removal (#1) and the beginning of polymer degradation with the elimination of water molecules (#2). Furthermore, at 270 ${}^{\circ }$C, the PVA main chain degradation began (#3), with pyrolysis and degradation events verified between 320 ${}^{\circ }$C and 500 ${}^{\circ }$C (#4). Similar results to those shown in Figure 1 are reported in the literature [25,26], showing the PVA polymer matrix degradation.

**Figure 2:**
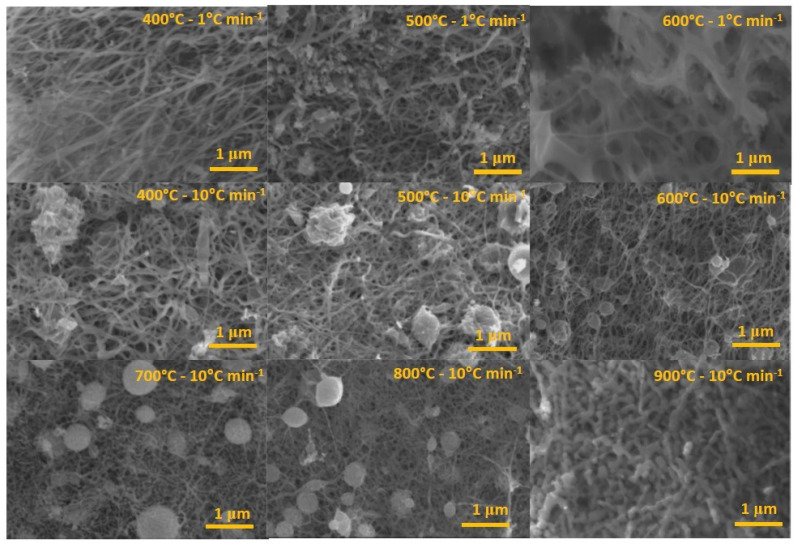
SEM images of the Nb${}_{2}$O${}_{5}$ fibers after the thermal treatments at different temperatures (400–900 ${}^{\circ }$C), using a heating rate of 1 ${}^{\circ }$C min${}^{-1}$ and 10 ${}^{\circ }$C min${}^{-1}$.

Once the PVA degradation behavior against heat treatment was verified, the morphology of the Nb${}_{2}$O${}_{5}$ fibers was analyzed. Thus, fibers were submitted in an annealing treatment from 400 ${}^{\circ }$C to 900 ${}^{\circ }$C for 2 h, with a fixed heating rate of 10 ${}^{\circ }$C min${}^{-1}$. The SEM images results (Figure 2) show the fiber format leakage and the consequent formation of the particles with the calcination temperature increasing. The predominance of fiber morphology is observed at temperatures from 400 ${}^{\circ }$C to 700 ${}^{\circ }$C, and agglomerates at higher temperatures. Additionally, the fiber average diameters obtained from 400 ${}^{\circ }$C to 700 ${}^{\circ }$C was 170, 140, 150, and 120 nm, respectively. However, when the temperature was raised to 800 ${}^{\circ }$C, the formation of a more significant number of shapeless particles with an average diameter of 135 nm was verified. Furthermore, the fiber loss format was observed after 900 ${}^{\circ }$C, which led to the particulate material formation.

The mentioned morphological characteristics are due to the increasing temperatures. Consequently, the energy supplied favors the Nb${}_{2}$O${}_{5}$ fiber coalescence effect, resulting in a fiber losing initial shape and introducing new particles, inducing growth until thermodynamic equilibrium [27,28]. Furthermore, according to Figure 2, samples treated at 400 ${}^{\circ }$C, 500 ${}^{\circ }$C, and 600 ${}^{\circ }$C using a heating rate of 1 ${}^{\circ }$C min${}^{-1}$ showed fiber-like morphology with mean diameters of 280, 130, and 84 nm, respectively. Thus, fibers obtained at 600 ${}^{\circ }$C showed greater homogeneity and smaller diameters when compared to those obtained at 10 ${}^{\circ }$C min${}^{-1}$. The obtained results are similar to the literature [29], which also verified morphological differences related to the heating rate during the annealing process; this step is essential for morphology formation. Besides that, at the lowest heating rate, a more regular aspect was defined for fibers without pores, attributed to greater control of polymeric matrix degradation.

**Figure 3:**
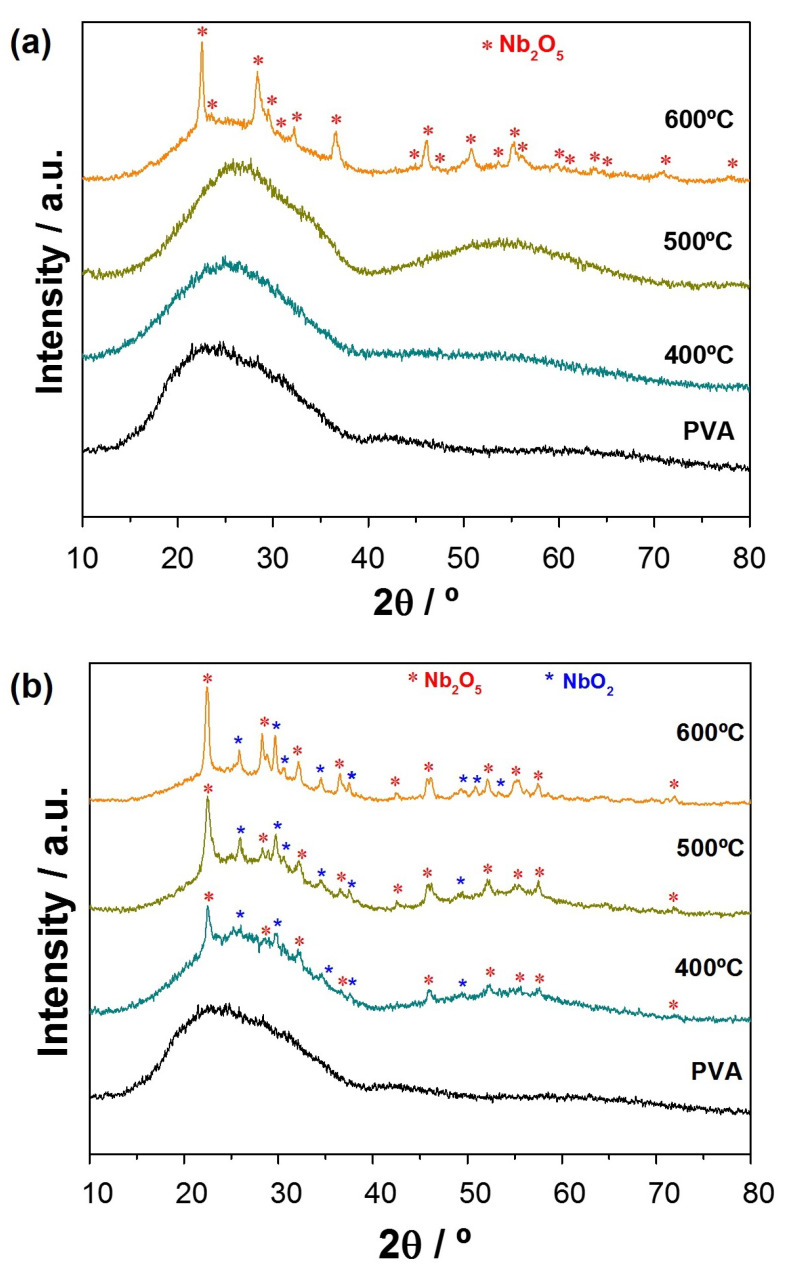
X-ray diffraction profiles of Nb${}_{2}$O${}_{5}$ fibers after thermal treatments at 400 ${}^{\circ }$C, 500 ${}^{\circ }$C, and 600 ${}^{\circ }$C under a heating rate of (**a**) 1 ${}^{\circ }$C min${}^{-1}$ and (**b**) 10 ${}^{\circ }$C min${}^{-1}$.

In order to verify the heating rate effects, the range of 400–600 ${}^{\circ }$C was chosen to observe the structural changes due to PVA matrix degradation and Nb${}_{2}$O${}_{5}$ calcination. Figure 3 shows the XRD diffractograms performed for Nb${}_{2}$O${}_{5}$ ceramic fibers. At this temperature, the lowest heating rate of 1 ${}^{\circ }$C min${}^{-1}$ (Figure 3a) contributed to the structural Nb${}_{2}$O${}_{5}$ phase formation that showed the orthorhombic phase characteristic peaks (JCPDS 27-1003). On the other hand, regarding the temperatures of 400 ${}^{\circ }$C and 500 ${}^{\circ }$C, the absence of the Nb${}_{2}$O${}_{5}$ peaks was observed, being detected as amorphous halos, similar to pure PVA. Thus, the temperature of 600 ${}^{\circ }$C was the minimum for the crystalline Nb${}_{2}$O${}_{5}$ phase formation.

In order to verify the effects of the heating rate on structural characteristics, the range of 400–600 ${}^{\circ }$C was chosen to observe the PVA matrix degradation impacts during the Nb${}_{2}$O${}_{5}$ calcination step. The diffractograms in Figure 3b prove the oxide formation at 400, 500, and 600 ${}^{\circ }$C, identifying the orthorhombic preferential T-Nb${}_{2}$O${}_{5}$ phase (JCPDS card n° 27-1003), besides the peaks of monoclinic M-Nb${}_{2}$O${}_{5}$ phase (JCPDS card n° 18-0910). In addition, the presence of a secondary NbO${}_{2}$ phase (JCPDS n° 01-082-1142) was also identified with more intense peaks located at 26.73°, 30.00°, and 49.62° (Figure 3b), presented in all temperature ranges from 400–600 ${}^{\circ }$C. The quantification of niobium phases in fibers heat-treated at 600 ${}^{\circ }$C for 10° min${}^{-1}$ was performed using Rietveld refinement, applying the inorganic crystal structure database (ICSD) as data input. The results showed 56% of T-Nb${}_{2}$O${}_{5}$ (ICSD 1840 = Pbam [30]), 15% of M-N${}_{2}$O${}_{5}$ (ICSD 16802 = B112/b [31]), and 29% of NbO${}_{2}$ (ICSD 75198 = P42/mnm [32]). The phases composition resulted in respective convergence and reliability values of Rwp and χ${}^{2}$ equal to 8.98% and 1.95, ensuring the veracity of the results.

By comparing the diffractograms obtained at the same heat treatment temperatures with a heating rate of 10 ${}^{\circ }$C min${}^{-1}$ and 1 ${}^{\circ }$C min${}^{-1}$ (Figure 3b), the lower rate decreased the polymeric degradation rate and favored a more controlled thermal behavior since the organic matrix elimination occurred and the structural arrangement formation of the ceramic fibers occurred. Furthermore, the heating rate faster increased degradation of the PVA fiber, changing the atmosphere with high CO${}_{2}$ elimination and reducing the atmosphere’s oxidizing capacity.

Studies indicate nanofibers are formed by nanograins that coalesce and grow when subjected to high calcination temperatures for long periods. For instance, ZnO nanofibers [33] submitted to elevated temperatures in extended periods generally result in superior crystallinity and grain sizes. The presence of grain boundaries makes nanofibers much more promising than monocrystalline nanowires or nanorods since grain boundaries play an important role in amplifying the resistance during the adsorption and desorption of gaseous species. This behavior is correlated with the present work since the minimum calcination temperature of 600 ${}^{\circ }$C contributed to structural nanofibers with properties that can be more significant for CO${}_{2}$ photocatalysis due to electron generation attributed to the relation with the grain boundary.

**Figure 4:**
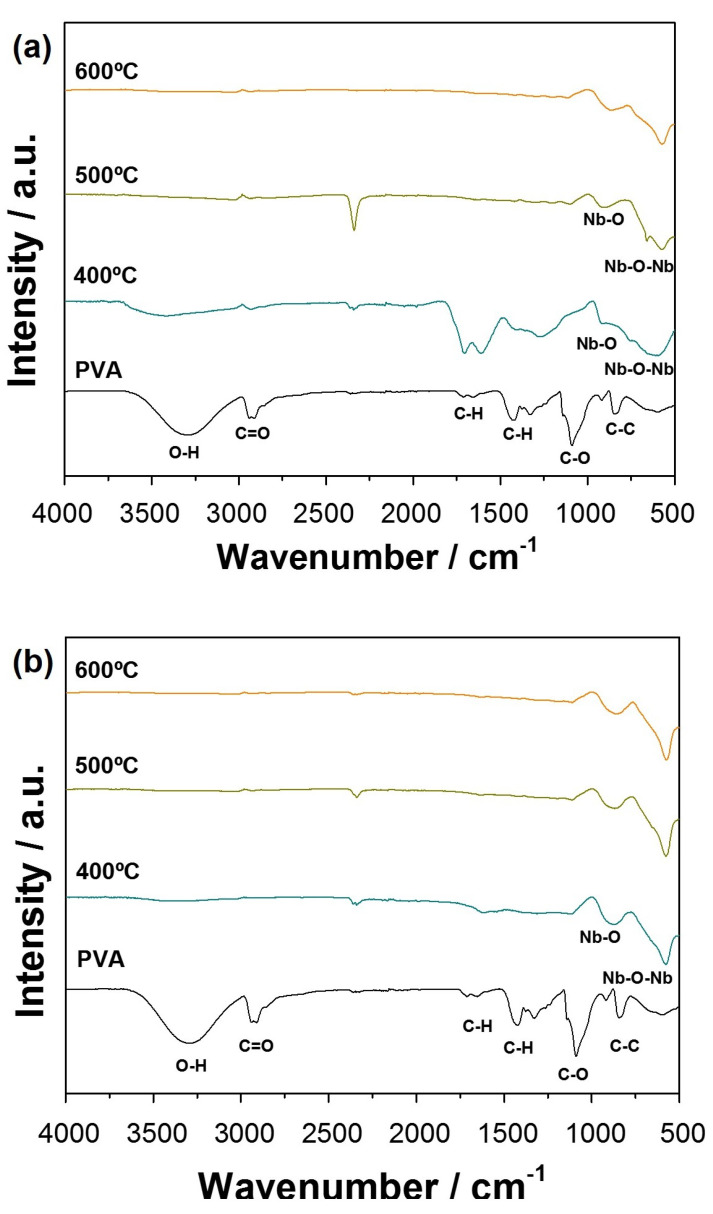
FTIR profiles of PVA and Nb${}_{2}$O${}_{5}$ ceramic fibers after thermal treatments at 400 ${}^{\circ }$C, 500 ${}^{\circ }$C or 600 ${}^{\circ }$C at a heating rate of (**a**) 1 ${}^{\circ }$C min${}^{-1}$ and (**b**) 10 ${}^{\circ }$C min${}^{-1}$.

In order to further understand the structural properties of the ceramic nanofibers, FTIR analysis was performed for different heating rates to identify the presence of residual organic material in the samples after annealing. Figure 4a shows the results of the FTIR analysis for the fibers calcined at a heating rate of 1 ${}^{\circ }$C min${}^{-1}$. Only the highest temperature of 600 ${}^{\circ }$C indicated the presence of pure Nb${}_{2}$O${}_{5}$ bands located at 872 cm${}^{-1}$ referring to bonds (Nb=O) and in 613 cm${}^{-1}$ associated with the bonds (Nb-O-Nb) [34,35]. The PVA polymeric matrix presented vibration bonds located in positions at 3314 cm${}^{-1}$ and 3332 cm${}^{-1}$ (O-H), 2917 cm${}^{-1}$ (C=O), 1500 cm${}^{-1}$ (C-H), 1000 cm${}^{-1}$ (C-O), and around 800 cm${}^{-1}$ (C-C) [36]. The same result is observed at temperatures of 400 ${}^{\circ }$C and 500 ${}^{\circ }$C. The event at 2500 cm${}^{-1}$ is attributed to the adsorption of CO${}_{2}$ molecules on semiconductors’ surfaces. Thus, the band intensity variation can be attributed to the change in the CO${}_{2}$ level present inside the room since the laboratory door remained open during the analysis period.

For the fibers spectra obtained at the rate of 10 ${}^{\circ }$C min${}^{-1}$ (Figure 4b), it is verified that even at the lowest temperatures of 400 ${}^{\circ }$C and 500 ${}^{\circ }$C, there is only the appearance of typical Nb-O bands. These results corroborate the data obtained from the XRD diffractograms (Figure 3), indicating that a minor decrease in the polymeric part elimination occurred when a lower heating rate was used, preventing the crystalline ceramic fiber formation at lower temperatures.

The fibers calcined at 1 ${}^{\circ }$C min${}^{-1}$ are different from those treated at 10 ${}^{\circ }$C min${}^{-1}$, especially for the 400 ${}^{\circ }$C, showing that the vibration modes of organic molecules are much more evident. However, with the heating rate of 10 ${}^{\circ }$C min${}^{-1}$, the fibers still have organic residues attributed to the rapid decomposition. Thus, these arrangements were not well organized, influencing the inexistence of some vibrational modes. In this way, decreasing the heating rate allows the samples to be exposed to the temperature for more extended periods, leading to the highest structural organization. In order to evaluate the optical properties of the samples, a diffuse reflectance spectroscopy (DRS) analysis was performed. Both fibers calcined at 600 ${}^{\circ }$C presented a similar band gap between 3.6 (10 ${}^{\circ }$C min${}^{-1}$) and 3.8 eV (1 ${}^{\circ }$C min${}^{-1}$), values of which are close to the expected range reported in the literature for Nb${}_{2}$O${}_{5}$ [37].

After the structural and morphological characterization of Nb${}_{2}$O${}_{5}$ samples, the fibers obtained at 600 ${}^{\circ }$C for 1 ${}^{\circ }$C min${}^{-1}$ and 10 ${}^{\circ }$C min${}^{-1}$ were selected for the CO${}_{2}$ photoreduction. These systems indicated the complete elimination of the organic precursor and the formation of the niobium oxide phases.

### 3.2. Photocatalytic Conversion of CO${}_{2}$

Nb${}_{2}$O${}_{5}$ and Nb${}_{2}$O${}_{5}$:NbO${}_{2}$ fibers were selected to verify the photocatalytic activity effects on the gaseous CO${}_{2}$ photoconversion. The kinetic behavior indicates the CO concentration (μmol g${}^{-1}$) produced during 6 h for each photocatalyst system. The Nb${}_{2}$O${}_{5}$ fibers produced more CO, increasing from 2.0 μmol g${}^{-1}$ (1 h) to 8.2 μmol g${}^{-1}$ after 6 h. On the other hand, the Nb${}_{2}$O${}_{5}$:NbO${}_{2}$ photocatalyst produced less CO, reaching 3.8 μmol g${}^{-1}$ in 6 h of reaction. This value was 46% lower than pure Nb${}_{2}$O${}_{5}$ in the same period to produce CO. Thus, pure Nb${}_{2}$O${}_{5}$ is a better photocatalyst for CO production, being the NbO${}_{2}$ (second phase) prejudices the photocatalytic system performance for CO${}_{2}$ photoreduction.

**Figure 5:**
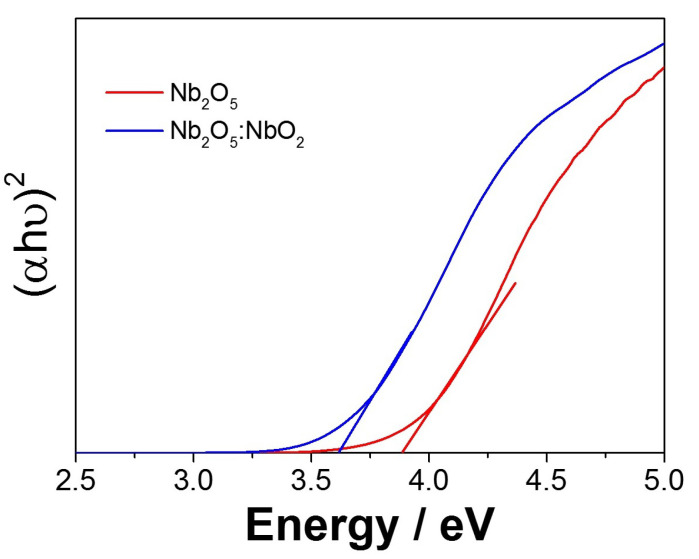
Diffuse reflectance spectroscopy (DRS) of fibers annealed at 600 ${}^{\circ }$C for 2 h, with heating rates of 1 and 10 ${}^{\circ }$C min${}^{-1}$.

The catalytic properties are strongly influenced by the presence of acidic and basic sites on the catalyst surface. Considering this behavior and according to the Lewis theory, Nb${}^{5+}$ cations have electron acceptor potential, acting as an acid, while O${}^{2-}$ anions act like a basic receiver. Therefore, the acid–base interactions that occur on the surface of Nb${}_{2}$O${}_{5}$ and the CO${}_{2}$ reaction can influence the formation of activated complexes. The NbO${}_{2}$ second phase presents a narrow band gap of about 0.7 eV and possesses an electronic configuration different from Nb${}_{2}$O${}_{5}$ (4d${}^{1}$ and 4d${}^{0}$, respectively). The electron presented in the 4d electronic layer can be donated or shared, facilitating the oxygen reduction reactions and, consequently, decreasing the oxidation number of the NbO${}_{2}$ compound, which can influence the decrease in photocatalytic activity [38]. Thus, despite obtaining a material with a lower band gap value, Nb${}_{2}$O${}_{5}$:NbO${}_{2}$ (3.3 eV) compared to Nb${}_{2}$O${}_{5}$ (3.8 eV), as seen in Figure 5, the second phase can promote a higher recombination rate of the photogenerated pairs, strongly influencing the photocatalytic properties.

**Figure 6:**
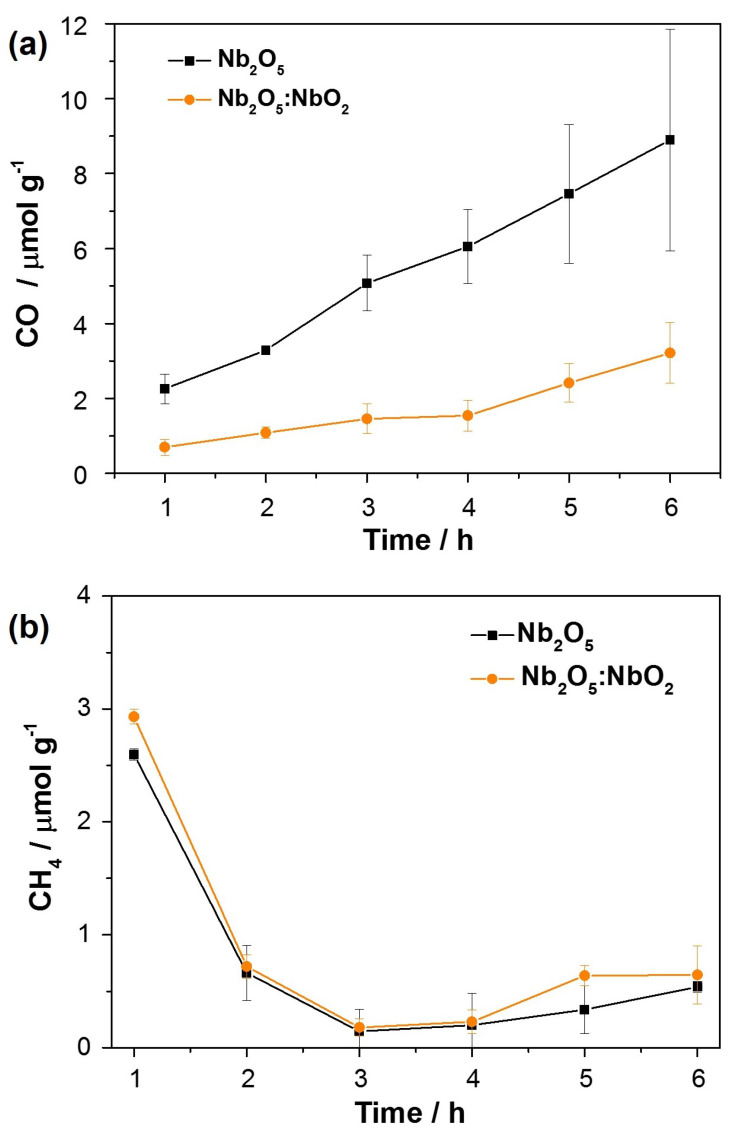
CO${}_{2}$ gaseous photoreduction in (**a**) CO and (**b**) CH${}_{4}$ during 6 h under UV-C irradiation.

Figure 6b shows the CH${}_{4}$ production by Nb${}_{2}$O${}_{5}$ and Nb${}_{2}$O${}_{5}$:NbO${}_{2}$ photocatalysts for 6 h. In the first hour of analysis, a CH${}_{4}$ amount of 2.6 and 2.9 μmol g${}^{-1}$ was photogenerated by Nb${}_{2}$O${}_{5}$ and Nb${}_{2}$O${}_{5}$:NbO${}_{2}$, respectively. However, the CH4 concentration dropped abruptly in the second hour, remaining practically constant (around 0.6 and 0.7 μmol g${}^{-1}$) for both materials. In this sense, it was observed that the different systems prepared based on niobium tend to produce CO preferentially. This behavior is justified because the CO${}_{2}$ reduction mechanism requires two electrons to produce CO molecules and eight electrons for CH${}_{4}$ formation. For this reason, the generation of CH${}_{4}$ is much more complex [39]. Furthermore, CO can strongly be adsorbed and desorbed on the photocatalyst surface in many cycles, affecting the yield of the CH4 formation significantly [40].

Studies reveal that the surface acidity of the photocatalyst can influence the selectivity of the products generated from CO${}_{2}$ photoreduction [11,12]. The size of nanoparticles and composition of the photocatalysts can also influence the selectivity during the photoreduction reaction [41]. Nogueira et al. [4] proposed an interaction mechanism between the surface of Nb${}_{2}$O${}_{5}$ with CO${}_{2}$ and the selectivity of products. The Nb${}_{2}$O${}_{5}$ structure is composed of NbO${}_{4}$ tetrahedral, forming NbO${}_{4}$-H${}_{2}$O structures in the presence of water vapor, which start to act as Lewis acid sites, receiving the oxygen electron pair from CO${}_{2}$, enabling coordination with the photocatalyst surface. Similarly, the preferential CO production for the samples is due to the high oxygen interaction between the CO${}_{2}$ molecules and photocatalyst active sites.

**Figure 7:**
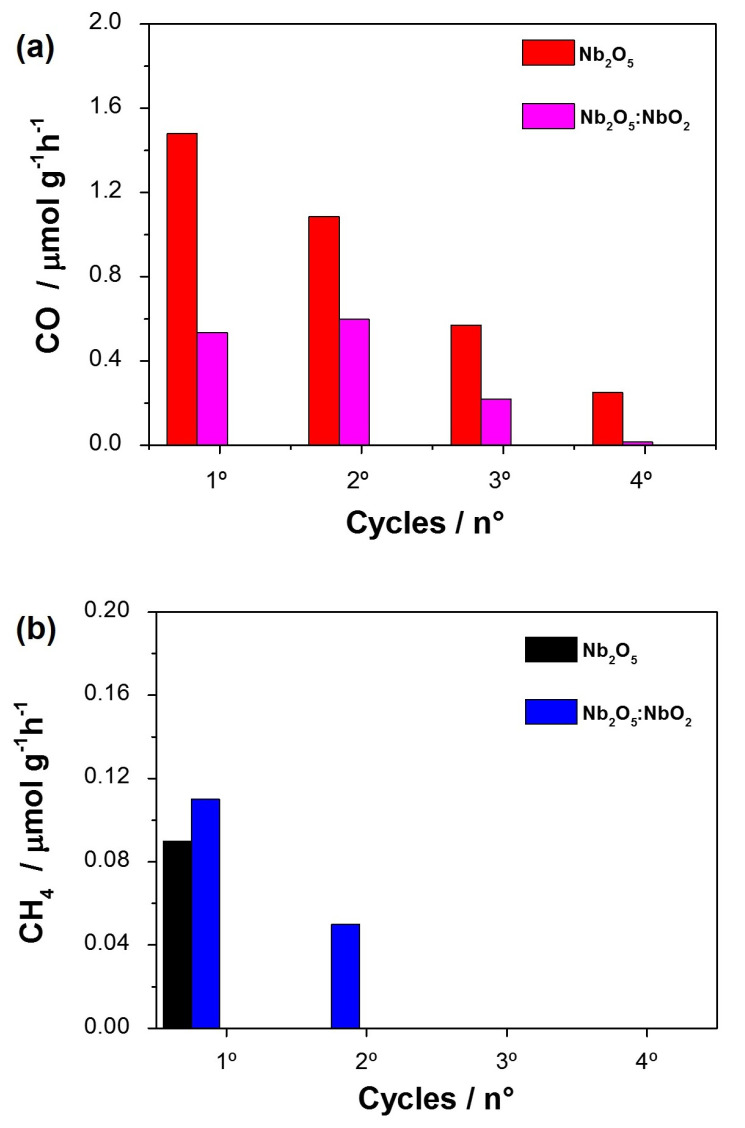
CO${}_{2}$ conversion rate into (**a**) CO and (**b**) CH${}_{4}$ products after a period of 6 h under UV-C irradiation, during four consecutive cycles.

After the first photocatalysis cycle of Nb${}_{2}$O${}_{5}$ and Nb${}_{2}$O${}_{5}$:NbO fibers, 3 reuse tests were performed to evaluate the stability of the samples. Figure 7 shows the production rate (μmol g${}^{-1}$h${}^{-1}$) of CO and CH${}_{4}$ per cycle and a decrease in the formation of both products over the consecutive cycles.

In Figure 7a, it is observed that Nb${}_{2}$O${}_{5}$ produced the highest CO concentration in all analysis cycles, being the only one that promoted significant amounts up to the 4th cycle. However, the Nb${}_{2}$O${}_{5}$:NbO${}_{2}$ system, despite the lower yield, remained stable in the CO production, preserving the photocatalytic activity until the third cycle in a relevant way. Thus, the second phase of the NbO${}_{2}$ indicated a lower degree of deactivation of the catalytic sites than pure Nb${}_{2}$O${}_{5}$. The deactivation of catalytic sites is one of the fundamental parameters for CO${}_{2}$ reduction [42]. In this sense, the Nb${}^{4+}$ oxidation state from NbO${}_{2}$ under O${}_{2}$ deficiency allows the formation of substitution defects in the Nb${}_{2}$O${}_{5}$ structure. Additionally, reducing Nb${}^{5+}$ to Nb${}^{4+}$ can influence the electron production in the catalytic sites and favor the formation of products, such as CH${}_{4}$ before the site deactivation from the niobium oxidation to the 5+ state [43].

Another factor to be highlighted is the photocatalytic stability of the synthesized materials since the production rates of both CO and CH${}_{4}$ decreased over time in new cycles. This behavior can be related to works involving Nb${}_{2}$O${}_{5}$ nanoparticles in the literature [12,44] since the photocatalysts presented a similar performance concerning reuse stability in CO production. Oliveira et al. [44] found a decrease of more than 50% in the CO production rate after four consecutive reuses using Nb${}_{2}$O${}_{5}$ nanoparticles, corroborating the present work results. According to the authors, a significant reduction in the CO reaction rate after the first cycle is attributed to the decrease of Nb-O groups on the surface due to the adsorption of CO${}_{2}$ molecules. This result is correlated with what is shown in Figure S1 (supplementary). Thus, it is possible to verify the increase of CO${}_{2}$ vibrational bands (#1) in the FTIR spectrum after the first cycle of the photoreduction process.

Additionally, Figure 7b shows that the Nb${}_{2}$O${}_{5}$:NbO${}_{2}$ system produced CH${}_{4}$ in a second reuse, maintaining about 50% of the amount observed in the previous cycle. This behavior was due to preserving the photocatalytic activity of the intermediate CO (see Figure 7a). Thus, although the NbO${}_{2}$ second phase in the Nb${}_{2}$O${}_{5}$ system decreases the overall photocatalytic performance, its presence enhanced CH${}_{4}$ production capacity. Therefore, the specific products’ production rate and the photocatalytic performance maintenance in the reuse cycles are directly related to the niobium oxide phases and active site preservation on the semiconductor surface.

## 4. Conclusions

The optimization of calcination parameters showed Nb${}_{2}$O${}_{5}$ fibers with structural and morphology control. The use of different heating rates, 1 and 10 ${}^{\circ }$C min${}^{-1}$, induced the formation of two photocatalysts, Nb${}_{2}$O${}_{5}$ and Nb${}_{2}$O${}_{5}$:NbO${}_{2}$, respectively. It was also verified that the annealing temperature influences fibers or particle phase formation and morphology. The CO${}_{2}$ photoreduction was more efficient Nb${}_{2}$O${}_{5}$ than over Nb${}_{2}$O${}_{5}$:NbO${}_{2}$, but both generated CO and CH${}_{4}$. Despite the lower photoreduction yield, a secondary NbO${}_{2}$ phase allowed a photocatalytic performance with better preservation. Therefore, ceramic nanofibers based on niobium oxide were effectively obtained, presenting CO${}_{2}$ photocatalytic conversion into commercial compounds.

## Data Availability

Not applicable.

## Supplementary Materials

The following are available online at https://www.mdpi.com/article/10.3390/nano11123268/s1, Figure S1. FTIR spectrum samples before and after the CO${}_{2}$ photoreduction process.

## References

[1] Malafatti J.O.D., Moreira A.J., Sciena C.R., Silva T.E.M., Freschic G.P.G., Pereira E.C., Paris E.C. (2020). Prozac® removal promoted by HAP:Nb_2_O_5_ nanoparticles system: By products, mechanism, and cytotoxicity assessment. J. Environ. Chem. Eng..

[2] Paris E.C., Malafatti J.O.D., Sciena C.R., Junior L.F.N., Zenatti A., Escote M.T., Moreira A.J., Freschi G.P.G. (2020). Nb_2_O_5_ nanoparticles decorated with magnetic ferrites for wastewater photocatalytic remediation. Environ. Sci. Pollut. Res..

[3] Izadpanah Ostad M., Niknam Shahrak M., Galli F. (2021). Photocatalytic carbon dioxide reduction to methanol catalyzed by ZnO, Pt, Au, and Cu nanoparticles decorated zeolitic imidazolate framework-8. J. CO_2_ Util..

[4] Budzianowski W.M., Postawa K. (2017). Renewable energy from biogas with reduced carbon dioxide footprint: Implications of applying different plant configurations and operating pressures. Renew. Sustain. Energy Rev..

[5] Kočí K., Obalová L., Lacný Z. (2008). Photocatalytic reduction of CO_2_ over TiO_2_ based catalysts. Chem. Pap..

[6] Oliveira J.A., Nogueira A.E., Gonçalves M.C.P., Paris E.C., Ribeiro C., Poirier G.Y., Giraldi T.R. (2018). Photoactivity of N-doped ZnO nanoparticles in oxidative and reductive reactions. Appl. Surf. Sci..

[7] Wang H., Zhang L., Wang K., Sun X., Wang W. (2019). Enhanced photocatalytic CO_2_ reduction to methane over WO_3_·0.33H_2_O via Mo doping. Appl. Catal. B Environ..

[8] Zeng Z., Yan Y., Chen J., Zan P., Tian Q., Chen P. (2019). Boosting the Photocatalytic Ability of Cu_2_O Nanowires for CO_2_ Conversion by MXene Quantum Dots. Adv. Funct. Mater..

[9] Ye L., Deng Y., Wang L., Xie H., Su F. (2019). Bismuth-Based Photocatalysts for Solar Photocatalytic Carbon Dioxide Conversion. ChemSusChem.

[10] Lopes O.F., Paris E.C., Ribeiro C. (2014). Synthesis of Nb_2_O_5_ nanoparticles through the oxidant peroxide method applied to organic pollutant photodegradation: A mechanistic study. Appl. Catal. B Environ..

[11] Nogueira A.E., Silva G.T.S.T., Oliveira J.A., Lopes O.F., Torres J.A., Carmo M., Ribeiro C. (2020). CuO Decoration Controls Nb_2_O_5_ Photocatalyst Selectivity in CO_2_ Reduction. ACS Appl. Energy Mater..

[12] da Silva G.T.S.T., Nogueira A.E., Oliveira J.A., Torres J.A., Lopes O.F., Ribeiro C. (2019). Acidic surface niobium pentoxide is catalytic active for CO_2_ photoreduction. Appl. Catal. B Environ..

[13] Deng H., Xu F., Cheng B., Yu J., Ho W. (2020). Photocatalytic CO_2_ reduction of C/ZnO nanofibers enhanced by an Ni-NiS cocatalyst. Nanoscale.

[14] Xu F., Zhang J., Zhu B., Yu J., Xu J. (2018). CuInS_2_ sensitized TiO_2_ hybrid nanofibers for improved photocatalytic CO_2_ reduction. Appl. Catal. B Environ..

[15] Kang S., Im T., Koh M., Lee C.S. (2020). Facile fabrication of electrospun black titania nanofibers decorated with graphitic carbon nitride for the application of photocatalytic CO_2_ reduction. J. CO_2_ Util..

[16] Li F., Ruan S., Yin Y., Zhang N., Zhang H., Li C., Chen Y. (2018). Facile synthesis of MnWO_4_/WO_3_ electrospun nanofibers as high performance visible-light driven photocatalysts. Mater. Lett..

[17] Szilágyi I.M., Santala E., Heikkilä M., Pore V., Kemell M., Nikitin T., Teucher G., Firkala T., Khriachtchev L., Räsänen M. (2013). Photocatalytic properties of WO_3_/TiO_2_ core/shell nanofibers prepared by electrospinning and atomic layer deposition. Chem. Vap. Depos..

[18] Qi S., Zuo R., Liu Y., Wang Y. (2013). Synthesis and photocatalytic activity of electrospun niobium oxide nanofibers. Mater. Res. Bull..

[19] de Jesus E.T., Moreira A.J., Sá M.C., Freschi G.P.G., Joya M.R., Li M.S., Paris E.C. (2021). Potential of Nb_2_O_5_ nanofibers in photocatalytic degradation of organic pollutants. Environ. Sci. Pollut. Res..

[20] Dai Q., Yuan B., Guo M., Zhang K., Chen X., Song Z., Nguyen T.T., Wang X., Lin S., Fan J. (2020). A novel nano-fibriform C- modified niobium pentoxide by using cellulose templates with highly visible-light photocatalytic performance. Ceram. Int..

[21] Li G., Zhang X., Lu H., Yan C., Chen K., Lu H., Gao J., Yang Z., Zhu G., Wang C. (2019). Ethanol sensing properties and reduced sensor resistance using porous Nb_2_O_5_-TiO_2_ n-n junction nanofibers. Sens. Actuators B Chem..

[22] Du Y., Zhang S., Wang J., Wu J., Dai H. (2018). Nb_2_O_5_ nanowires in-situ grown on carbon fiber: A high-efficiency material for the photocatalytic reduction of Cr(VI). J. Environ. Sci..

[23] Kumari N., Gaurav K., Samdarshi S.K., Bhattacharyya A.S., Paul S., Rajbongshi B.M., Mohanty K. (2020). Dependence of photoactivity of niobium pentoxide (Nb_2_O_5_) on crystalline phase and electrokinetic potential of the hydrocolloid. Sol. Energy Mater. Sol. Cells.

[24] Nakane K., Morinaga M., Ogata N. (2013). Formation of niobium oxide and carbide nanofibers from poly(vinyl alcohol)/niobium oxide composite nanofibers. J. Mater. Sci..

[25] Reguieg F., Ricci L., Bouyacoub N., Belbachir M., Bertoldo M. (2020). Thermal characterization by DSC and TGA analyses of PVA hydrogels with organic and sodium MMT. Polym. Bull..

[26] Niazi M.B.K., Jahan Z., Ahmed A., Uzair B., Mukhtar A., Gregersen Ø.W. (2020). Mechanical and thermal properties of carboxymethyl fibers (CMF)/PVA based nanocomposite membranes. J. Ind. Eng. Chem..

[27] Ferreira E.d.P., Bessa L.P., Cardoso V.L., Reis M.H.M. (2019). Influence of sintering temperature on the morphology of ceramic hollow fibers prepared from niobium pentoxide. Int. J. Appl. Ceram. Technol..

[28] Nakhowong R. (2015). Fabrication and characterization of MnTiO_3_ nanofibers by sol-gel assisted electrospinning. Mater. Lett..

[29] Wan M., Zhu H., Zhang S.G., Jin H.N., Wen Y.K., Wang L.N., Zhang M., Du M.L. (2018). Building block nanoparticles engineering induces multi-element perovskite hollow nanofibers structure evolution to trigger enhanced oxygen evolution. Electrochim. Acta.

[30] Kato K., Tamura S.Y. (1975). Die kristallstruktur von T-Nb_2_O_5_. Acta Crystallogr. Sect. B Struct. Crystallogr. Cryst. Chem..

[31] Laves F., Petter W., Wulf H. (1954). Die Kristallstruktur von *ζ*-Nb_2_O_5_. Naturwissenschaften.

[32] Bolzan A.A., Fong C., Kennedy B.J., Howard C.J. (1994). A powder neutron diffraction study of semiconducting and metallic niobium dioxide. J. Sol. Stat. Chem..

[33] Katoch A., Sun G.J., Choi S.W., Byun J.H., Kim S.S. (2013). Competitive influence of grain size and crystallinity on gas sensing performances of ZnO nanofibers. Sens. Actuators B Chem..

[34] Aziz S.B., Brza M.A., Hamsan M.H., Kadir M.F.Z., Muzakir S.K., Abdulwahid R.T. (2020). Effect of ohmic-drop on electrochemical performance of EDLC fabricated from PVA:dextran:NH_4_I based polymer blend electrolytes. J. Mater. Res. Technol..

[35] Hass Caetano Lacerda E., Monteiro F.C., Kloss J.R., Fujiwara S.T. (2020). Bentonite clay modified with Nb_2_O_5_: An efficient and reused photocatalyst for the degradation of reactive textile dye. J. Photochem. Photobiol. A Chem..

[36] Rad L.R., Momeni A., Ghazani B.F., Irani M., Mahmoudi M., Noghreh B. (2014). Removal of Ni^2+^ and Cd^2+^ ions from aqueous solutions using electrospun PVA/zeolite nanofibrous adsorbent. Chem. Eng. J..

[37] Sin J.C., Chin Y.H., Lam S.M. (2019). Wo_3_/nb_2_o_5_ nanoparticles-decorated hierarchical porous zno microspheres for enhanced photocatalytic degradation of palm oil mill effluent and simultaneous production of biogas. Key Eng. Mater..

[38] Peng Y., Lin C., Tang M., Yang L., Yang Y., Liu J., Huang Z., Li Z. (2020). Niobium pentoxide ultra-thin nanosheets: A photocatalytic degradation and recyclable surface-enhanced Raman scattering substrate. Appl. Surf. Sci..

[39] Inoue T., Fujishima A., Konishi S., Honda K. (1979). Photoelectrocatalytic reduction of carbon dioxide in aqueous suspensions of semiconductor powders [3]. Nature.

[40] Raza A., Shen H., Haidry A.A., Sun L., Liu R., Cui S. (2020). Studies of Z-scheme WO_3_-TiO_2_/Cu_2_ZnSnS_4_ ternary nanocomposite with enhanced CO_2_ photoreduction under visible light irradiation. J. CO_2_ Util..

[41] Jia J., Wang H., Lu Z., O’Brien P.G., Ghoussoub M., Duchesne P., Zheng Z., Li P., Qiao Q., Wang L. (2017). Photothermal Catalyst Engineering: Hydrogenation of Gaseous CO_2_ with High Activity and Tailored Selectivity. Adv. Sci..

[42] Wang S., Han X., Zhang Y., Tian N., Ma T., Huang H. (2021). Inside-and-Out Semiconductor Engineering for CO_2_ Photoreduction: From Recent Advances to New Trends. Small Struct..

[43] Mokrushin A.S., Simonenko T.L., Simonenko N.P., Gorobtsov P.Y., Kadyrov N.C., Simonenko E.P., Sevastyanov V.G., Kuznetsov N.T. (2021). Chemoresistive Gas-Sensing Properties of Highly Dispersed Nb_2_O_5_ Obtained by Programmable Precipitation. J. Alloys Compd..

[44] Oliveira J.A., Torres J.A., Gonçalves R.V., Ribeiro C., Nogueira F.G.E., Ruotolo L.A.M. (2021). Photocatalytic CO_2_ reduction over Nb_2_O_5_/basic bismuth nitrate nanocomposites. Mater. Res. Bull..

