# Supplementary figures and images for: Preparation and Application of Nb2O5 Nanofibers in CO2 Photoconversion
Source: Nanomaterials (Basel). 2021 Dec 1;11(12):3268. doi: 10.3390/nano11123268 (PMC8704612; doi:10.3390/nano11123268)

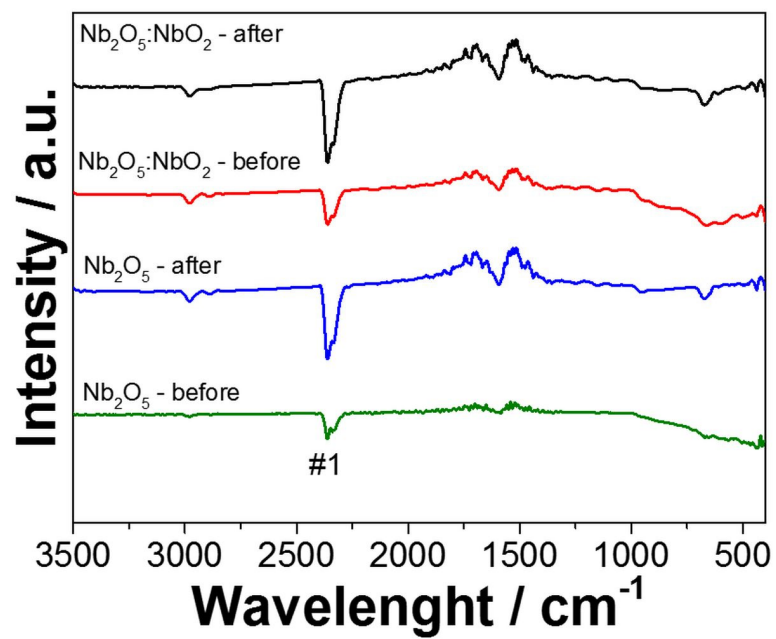

**Figure S1.** FTIR spectrum samples before and after the CO<sub>2</sub> photoreduction process.

Supplement: Supplementary file 1 [file nanomaterials-11-03268-s001.zip › nanomaterials-1450153-supplementary.pdf]
